# Developing an Intelligent Data Analysis Approach for Marine Sediments

**DOI:** 10.3390/molecules27196539

**Published:** 2022-10-03

**Authors:** Miroslava Nedyalkova, Vasil Simeonov

**Affiliations:** 1 Department of Inorganic Chemistry, Faculty of Chemistry and Pharmacy, University of Sofia “St. Kl. Ohridski”, 1164 Sofia, Bulgaria; 2 Department of Analytical Chemistry, Faculty of Chemistry and Pharmacy, University of Sofia “St. Kl. Ohridski”, 1164 Sofia, Bulgaria

**Keywords:** intelligent data analysis, marine sediments, pollution sources, source apportionment, PCA, anthropogenic events

## Abstract

(1) Background: As the chemical and physicochemical properties of marine sediments are closely related to natural and anthropogenic events, it is a real challenge to use their specific assessment as an indicator of environmental pollution discharges. (2) Methods: It is addressed in this study that collection with intelligent data analysis methods, such as cluster analysis, principal component analysis, and source apportionment modeling, are applied for the assessment of the quality of marine sediment and for the identification of the contribution of pollution sources to the formation of the total concentration of polluting species. A study of sediment samples was carried out on 174 samples from three different areas along the coast of the Varna Gulf, Bulgaria. This was performed to determine the effects of pollution. As chemical descriptors, 34 indicators (toxic metals, polyaromatic hydrocarbons, polychlorinated biphenyls, nutrient components, humidity, and ignition loss) were used. The major goal of the present study was to assess the sediment quality in three different areas along the Gulf of Varna, Bulgaria by the source apportionment method. (3) Results: There is a general pattern for identifying three types of pollution sources in each area of the coastline with varying degrees of variation between zone A (industrially impacted zones), zone B (recreational areas), and zone C (anthropogenic and industrial wastes). (4) Conclusions: The quantitative apportionment procedure made it possible to determine the contribution of each identified pollution source for each zone in forming the total pollutant concentrations.

## 1. Introduction

The factors affecting the chemical and physicochemical properties of marine sediments are closely related to various natural and anthropogenic events, and it is a real scientific challenge to use their specific assessment as a convenient mode of indicating different sides of environmental pollution discharges. There are various studies that attempt to evaluate the impact of different factors on marine sediment quality using classical univariate statistical methods such as regression or correlation analysis, where the purpose is to find reliable links between chemical or physicochemical monitoring values each other or related to indicators such as depth, geographical location or vicinity to pollution sources [1,2,3,4,5,6,7]. In most of these regression or correlation studies, the basic goal is after the selection of important tracers to point out the relationship between sediment composition and the sediment morphologic features or diagenesis [8,9,10]. It is critical to note that selecting specific sediment composition tracers is not a strict rule or routine. In fact, it may be considered a more practical option based on the level of concentration levels, the fact that it will remain a constant presence in the sediment bulk, and, more importantly, the fact that it will be unaffected by anthropogenic pollution sources. Specific tracers for general studies not related to apportionment of pollution sources and sediment quality assessment are iron, aluminum, total organic carbon (TOC) content, and sediment grain size. Nevertheless, a classical model suggested by Daskalakis and O’Connor [8], known as the “baseline model”, estimates the impact of anthropogenic factors with iron as a tracer component.

In the context of marine sediment composition and dynamics, such a simple univariate approach has many limitations and often ignores the computation of mixed interactions. The application of multivariate statistical methods (chemometrics or intelligent data analysis) makes it possible to interpret, classify and model the monitoring data sets from different geographical sediment sampling locations and sample collection periods. In several recently published studies [11,12,13,14,15,16,17,18,19,20,21,22,23,24,25,26,27,28], a small selection of numerous publications dedicated to the chemometric interpretation of sediment monitoring data, the whole spectrum of intelligent data analysis methods is involved. However, the references cited are representative of the opportunities offered by chemometrics (supervised and non-supervised pattern recognition methods, projection methods, dimension reduction methods, apportionment strategies, self-organizing maps approach, etc.). The machine learning methods are applied for assessing sediment quality, pollution risk assessment, apportionment of the contribution of identified pollution sources to the total concentration of sediment chemical composition, etc.

We use a collection of marine surface sediments from the coastline of the Black Sea (Gulf of Varna, Bulgaria) using chemometrics methods to estimate the sediment quality in three different zones at the sampling area. It will be of substantial practical interest to perform pollution source apportionment for each of the zones studied. This will enable us to compare the risk of pollution in recreation zones, industrial coastal zones, and zones with intensive traffic. It may also be possible to discern which specific descriptors are associated with each zone (cluster of sampling points) and whether these descriptors are related only to anthropogenic influences or whether they are also caused by natural influences.

## 2. Results and Discussion

### 2.1. Input Data Set

**Figure 1:**
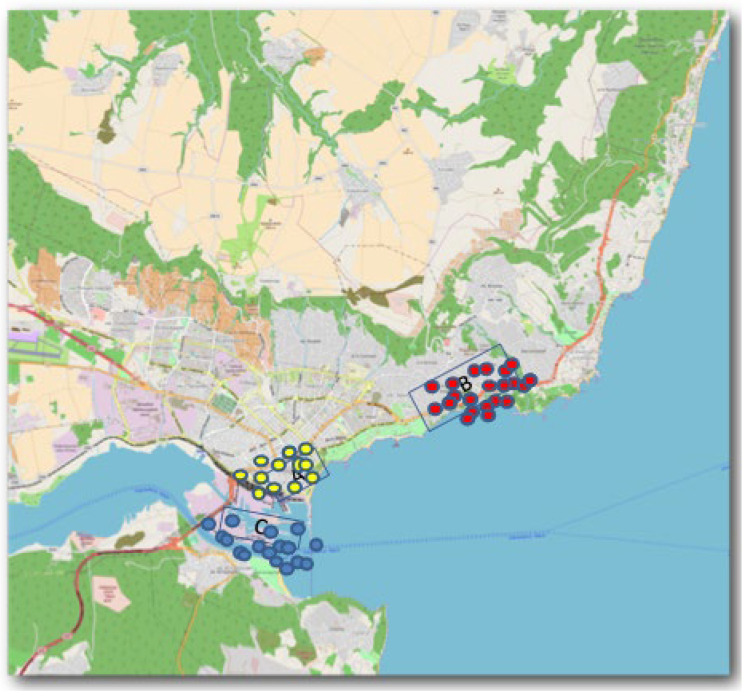
Sampling area for the explored zones A, B and C.

The data set consists of 174 marine sediment samples collected from three different zones of the coastal area of the Gulf of Varna, the Black Sea, Bulgarian aquatory named conditionally zone A, zone B, and zone C (Figure 1). The monitoring analysis was performed for 46 parameters. Still, due to some limitations (missing data, lack of any variability for some parameters), the final number of parameters was 34. The sampling was performed at a distance of 1 km from shore.

As already mentioned, the data set was offered by the coworkers of the Department of Marine Chemistry, Bulgarian Academy of Science and the authors were not engaged in sampling and sampling analysis. The choice of the variables included in the data set was no responsibility of the authors. Several of the parameters measured were at concentrations below their analytical limit of detection and, thus, eliminated from the variables list such as mercury, cadmium, etc. We were aware of the fact that the absence of some important variables (related to the crustal structure of sediments such as iron, aluminium, lithium, manganese, grain size, etc.) leads to some uncertain conclusions from the chemometric data interpretation.

Unfortunately, the grain size was not included in the data interpretation since it was not presented by the data producers. It could be stated that the variable “grain size” lacks variability and was a priori eliminated from the data set. The same consideration holds true for the choice of PCBs, Cd, Hg—only those PCBs (and, respectively, levels of Hg and Cd, which have significant variability were included in the data mining.

### 2.2. Chemometrics Results

Sampling area: Zone A [49 × 34]

Cluster analysis

**Figure 2:**
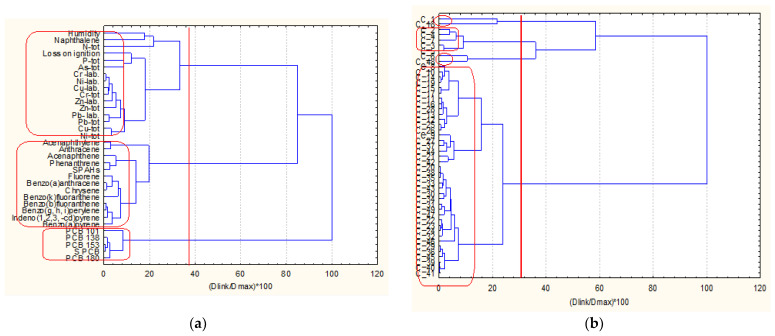
Hierarchical dendrogram (**a**) for clustering of 34 variables for zone A Description of what is contained in the first panel: (**b**) for clustering of 49 sampling locations.

The next two figures (Figure 2a,b) show the hierarchical dendrograms for clustering of the 49 objects (surface sediment sampling sites) and 34 variables (chemical parameters) for zone A.

Three major clusters of variables (Figure 2) are well defined. The first one (the bottom cluster on the dendrogram) includes all PCB variables and indicates the PCB pollution impact on the surface sediments. The second cluster (in the middle of the dendrogram) includes almost all PAH variables and shows the other pollution source composition in the surface sediments. Finally, the third group of similarities (the upper cluster in the dendrogram) proves the collective impact of metal pollution (both mobile and total concentrations. In this bigger cluster, the physicochemical indicators (humidity and loss of ignition) are included. This is evidence that these parameters are closely related rather to the metal pollution species than to the organic pollution components. The only exception is naphthalene, whose presence in this cluster is probably due to some local conditions. This is just a general assumption since no grain size data were available.

The sampling locations from zone A are clustered into four patterns of similarity. The biggest cluster (bottom group) consists of a total of 40 members, and the other three clusters are with a small number of sampling sites involved (2, 5, 2 from the bottom up to the top of the dendrogram). It could be assumed that the large cluster represents a homogeneous with respect to chemical composition and physicochemical properties area of sediment since the other three groups indicated specific locations with different properties as compared to those clustered in the large homogeneous pattern.

**Figure 3:**
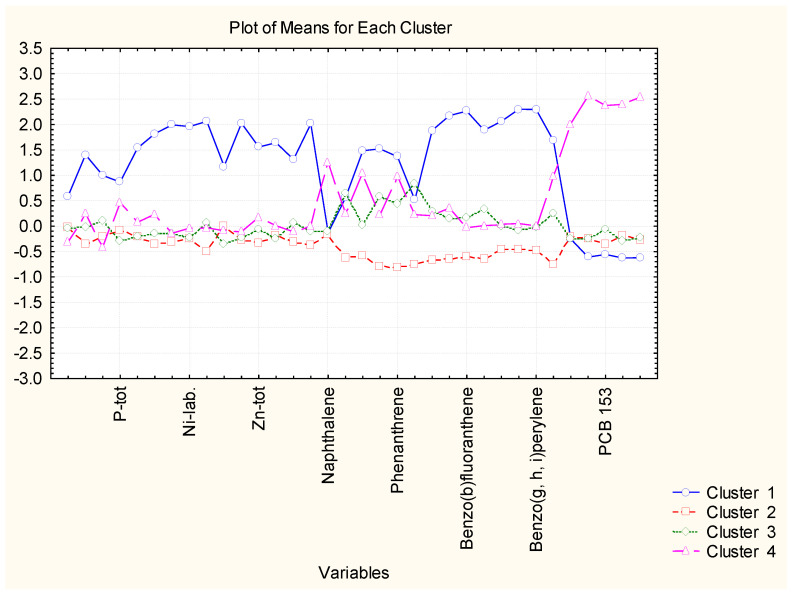
Plot of means for each variable for each identified cluster.

**Figure 4:**
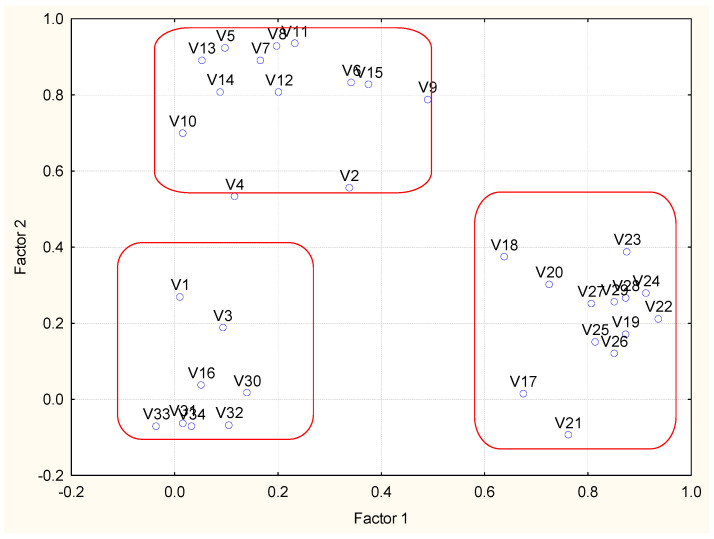
Biplot of factor loadings (Factor 1 vs. Factor 2 plane).

The nonhierarchical clustering (K-means clustering) into preliminary determined four clustering groups presented the same partitioning of the sediment sampling locations as in hierarchical cluster analysis. Still, it made it likely to readily determine the reason for the grouping. In that figure, the average values of each variable for each determined cluster are shown. Cluster 2 in Figure 3 is the cluster with 40 members; cluster 3 and cluster 4 contain two members each, and cluster 1–5 members. It is readily seen from Figure 4 that the concentration levels for the members of the biggest clusters are the lowest ones, and it could be presumed that this pattern of sediment sampling locations represents a relatively clean region for all groups of species—nutrition components, metals, PAHs and PCBs.

When we perform cluster analysis, the input raw data are standardized (z-transform) which is a standard approach to eliminate differences in variable dimensions and normalize the input data set (with a mean value of 0 and standard deviation of ±1). The averages for each variable for each one of the identified clusters are calculated on the transformed values (that is why they are either positive or negative). The variables presented on the horizontal axis are just part of the total list of all 34 variables but always in one and the same sequence (indicated in the Tables in the Supplementary Materials).

Cluster 1 indicates the grouping of locations with the highest values of pollutant concentrations except for PCBs. Therefore, this is a part of zone A with serious pollution by nutrients, metals, and PAHs. Since zone A is characteristic of the industrially impacted part of the Varna gulf, these five points included in cluster 2 are the most identified polluted sites due to their closer vicinity to cement and polymer production plants.

Clusters 3 and 4 contain just two members each and are characterized by slightly increased PAH levels (both partitioning) and the highest levels of PCB (cluster 4). The sites included in the clusters are also subject to organic pollution from industrial sources.

Moreover, factor analysis was conducted on the data set to better understand its structure. In Table 1, the factor loadings for 34 variables for four latent factors are presented. The Table 1 Factor loadings are presented in the Supplementary Materials.

Four latent factors explain 80% of the total variance of the system. The special location of the loadings is shown in Figure 4. Latent factors 1 and 2 explain almost one and the same percentage of the total variance and could be conditionally named “PAHs pollution factor” and “metals pollution factor”. The last one is correlated to the variable loss of ignition usually linked to the sediment metal content. These are the most significant latent factors for industrial coastal sediment pollution in zone A.

Latent factor 3 explains over 14% of the total variance and could be conditionally named “PCBs pollution factor”. More specific is the role of latent factor 4 (about 8% of the total variance of the system) and it reveals the relationship between sediment humidity and concentration of total nitrogen, The factor loadings for humidity are almost equal for factor 2 and 4, so it could be assumed that humidity is correlated to both metal content, loss of ignition and to nutrient species. This comparison requires carefulattention to the placement of local source of the pollutants. 

Pollution source apportionment for industrial zone A (absolute principal components score regression after Thurston-Spengler procedure). 

In Table 2, the results of the source apportionment procedure (in fact principal components regression analysis) are presented. In this table, the sums of PAHs and PCBs are included as variables along with total and labile metal concentrations. Nutrient components are also included but no apportionment is performed for the physicochemical descriptors.

Based on the results from multivariate analysis methods the contribution of each latent factor (pollution source) in % for forming the total concentration of each pollution species is given. The first column after the column with the variable names reveals the intercept of the regression model (usually associated with the unexplained contribution of unidentified pollution sources), and the last column gives the value of the multiple correlation coefficient (traditionally associated with the model fit). It can be figured that the regression models show a moderately good fit and could logically explain the contribution of each pollution source information in the total element concentration. Moreover, we also find that in most cases, the metal pollution is accompanied by PAHs and PCBs contributions due probably to technological process details for the industrial zone of the Varna gulf.

Sampling area: Zone B [105 × 34]

Cluster analysis

Zone B represents the coastal sediment composition and pollution impact of a typical recreation area where many beaches, hotels, camping places, and private lodgings are located. Although no active industrial activity is characteristic of the sampling area, anthropogenic activity and respective domestic and public activity pollution are possible. 

**Figure 5:**
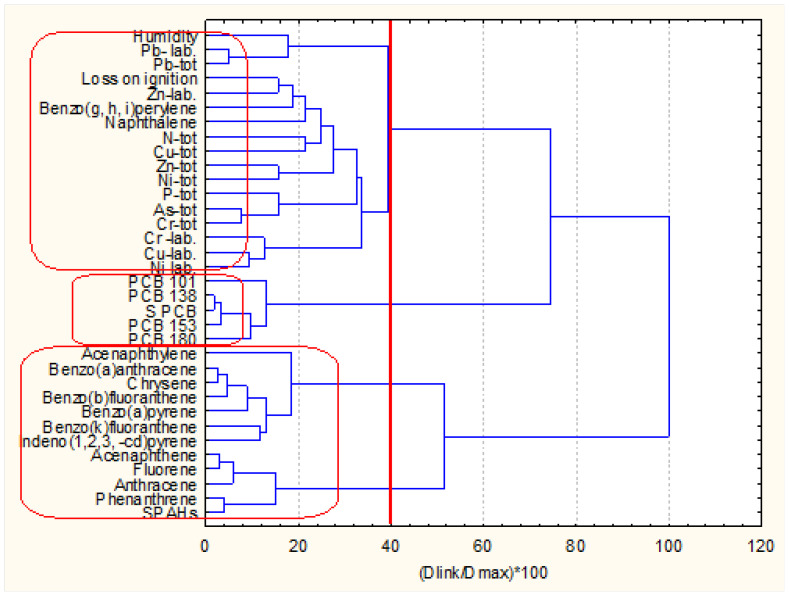
Hierarchical dendrogram for clustering of 34 variables for zone B.

**Figure 6:**
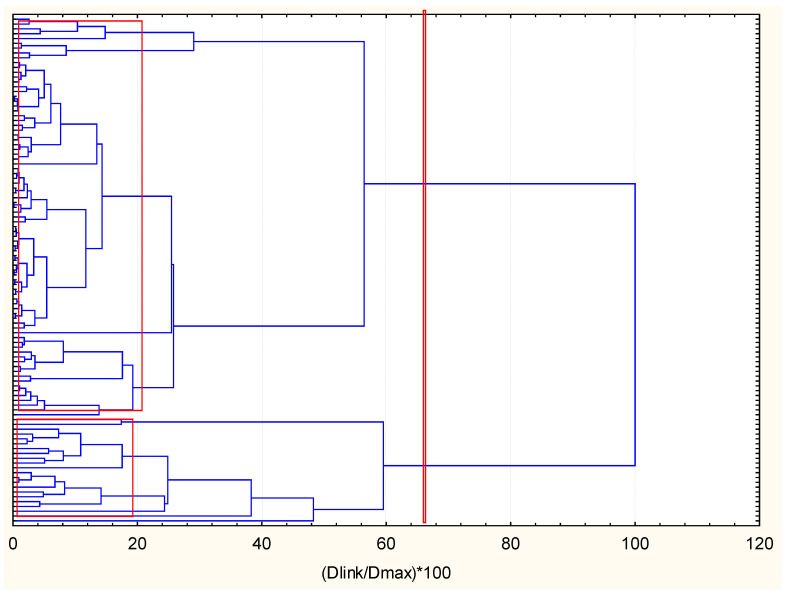
Hierarchical dendrogram for clustering of 105 sampling locations of zone B.

The next two figures (Figure 5 and Figure 6) represent the hierarchical dendrograms for clustering of the variables (chemical indicators) and objects (sampling locations) for zone B.

The linkage of the variables corresponds to that of the variables clustering for zone A. Very distinctive patterns in Figure 6 is the pattern of similarity for PCBs and PAHs. However, the biggest cluster for zone B variables partitioning contains the metals (labile and total form) and two PAHs—naphthalene and benzo (g, h, i) perylene. This signifies the possible common source of these PAH species and the metal pollutants (road traffic, domestic burning, etc.).

The clustering of the objects belonging to zone B (105 samples of coastal sediments) as shown in Figure 6 offers the interpretation of two major clusters—a big one with 88 members and a smaller one with 17 members. Both clusters are relatively homogeneous and the difference between them should be sought in the specific descriptors for both identified clusters.

In Figure 6, the average values for each variable for each one of the identified clusters (the configuration was proven by application of K-means clustering for a priori chosen number of clusters).

**Figure 7:**
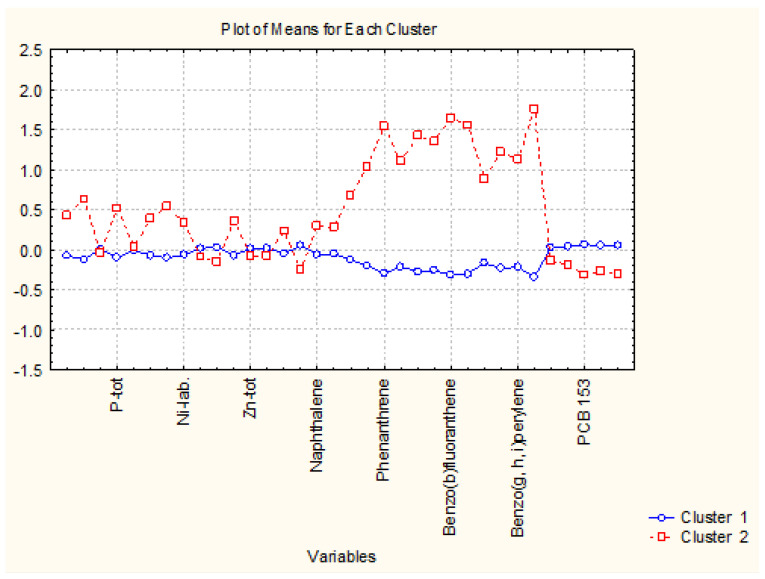
Plot of means for each variable for each identified cluster in zone B.

As indicated in Figure 7 cluster 1 (88 members) is characterized by low levels of almost all chemical parameters. Slightly higher values are observed for PCB values. It could be assumed that the locations included in cluster 1 are free of pollution. The other 17 locations (members of cluster 2) differ from those in cluster 1 with significantly higher levels of PAHs. Probably, these more polluted locations are subject mainly to enhanced traffic in the region being typical for residential areas with solid road nets.

For a more detailed clarification of the data, set structure factor analysis was carried out. The factor loadings for all 34 variables are presented in Table S2 in the Supplementary Materials.

Four latent factors explaining over 60% of the total variance are responsible for the data set structure. Latent factor 1 (explanation of 25.8 of the total variance) includes high and significant factor loadings for most PAHs. Still, a PAH group of significant loadings could also be detected on latent factor 4, and a single PAH species (benzo (g, h, i)perylene) shows high loading in latent factor 3. Naphthalene could not be attributed to any latent factor. This situation differs from the partitioning in zone A (very compact grouping of PAHs) and suggests the existence of different sources of PAHs in area B. Factor 1 could be conditionally named “traffic caused PAHs pollution”.

Latent factor 2 explains over 18% of the total variance and could be conditionally named PCBs pollution factor”. All PCB variables are showing significant factor loadings, which is an indication of a common source.

Latent factor 3 could be determined as a “metal pollution factor” (11.1% of the total variance). Significant factor loadings show the mobile forms of Zn, Cu, Ni, Pb and the total forms of Cr and Pb. The other metal species are characterized by statistically insignificant factor loadings and could not be related to specific pollution sources. In Factor 3, benzo (g, h, i)perylene is correlated to the metal species with significant loadings. Again, the sediment quality in coastal zone B is partially dependent on some specific sources related to pollution transfer from air and soil sources. 

Factor 4 is another conditional PAH pollution factor linked probably to the transfer of industrial wastes from zone A. 

It is important to note that some variables (humidity, loss of ignition, nutrition components, mobile form of Cr, total concentration of As, Zn, Cu, Ni) do not have statistically significant loadings and could not be attributed to some specific sources related to zone B. It indicates that the sediment quality in zone B is not dependent on local pollution sources, as is the case with zone A.

**Figure 8:**
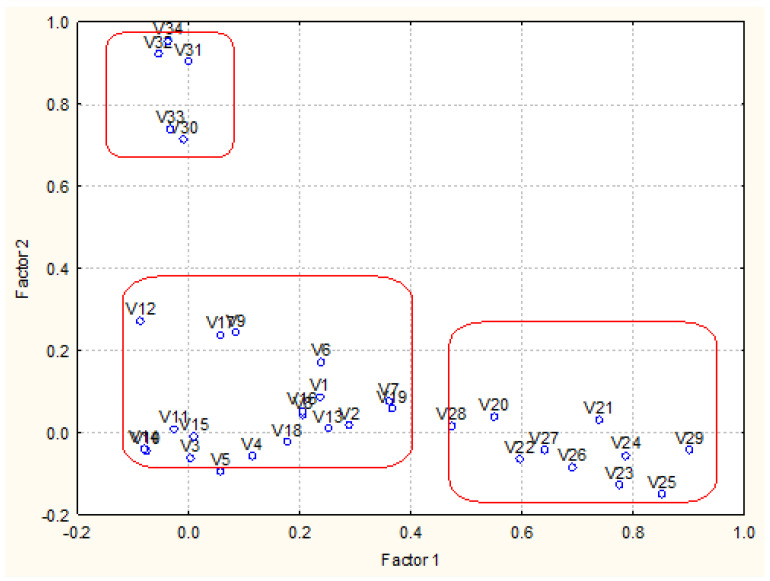
Biplot of factor loadings (Factor 1 vs. Factor 2 plane).

Figure 8 illustrates the grouping of variables with respect to their factor loadings. The separate factors are well presented (variables names decoded in the Supplementary Materials Table S3). In Table 3, the results of the source apportionment procedure are shown.

The APCS regression models describe quite satisfactorily the individual apportionment for zone B. Significant number of models is not adequate and cannot be used for model purposes. Therefore, in zone B, fewer components are responsible for proper modeling of the sediment quality. The dynamic processes of sedimentation and mass transfer in zone B are more intensive and important for sediment assessment.

Sampling area: Zone C [20 × 34]

Cluster analysis

**Figure 9:**
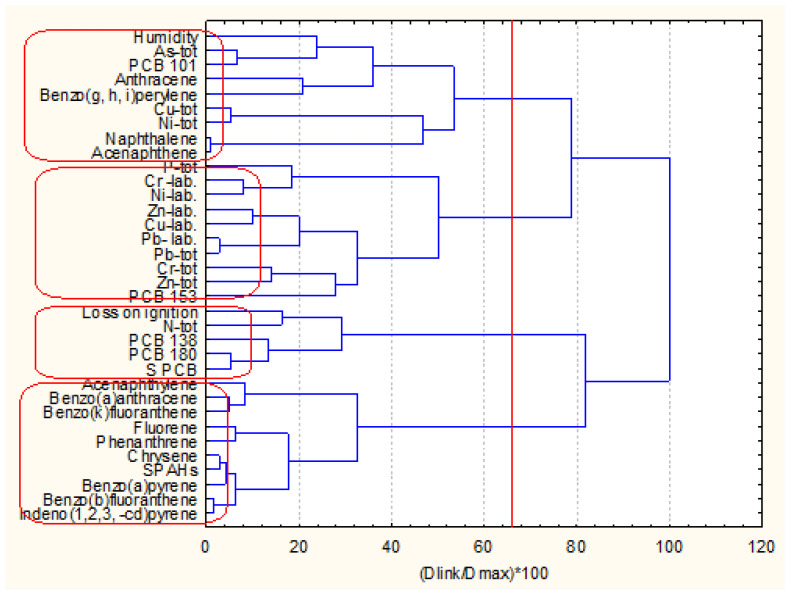
Hierarchical dendrogram for clustering of 34 variables for zone C.

**Figure 10:**
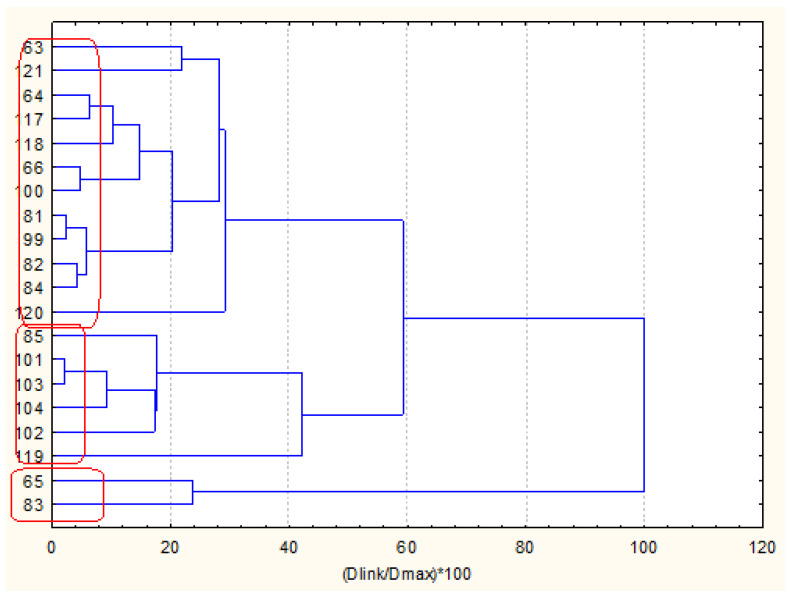
Hierarchical dendrogram for clustering of 20 objects for zone C.

In Figure 9 and Figure 10, the hierarchical dendrograms for clustering of 34 variables and 20 objects for zone C are presented.

The hierarchical clustering of the 20 sediment sampling sites in zone C reveals three major clusters of objects: cluster 1 with 12 objects (the upper cluster), cluster 2 with six objects (the middle cluster), and cluster 2 with only two members (the bottom cluster). 

**Figure 11:**
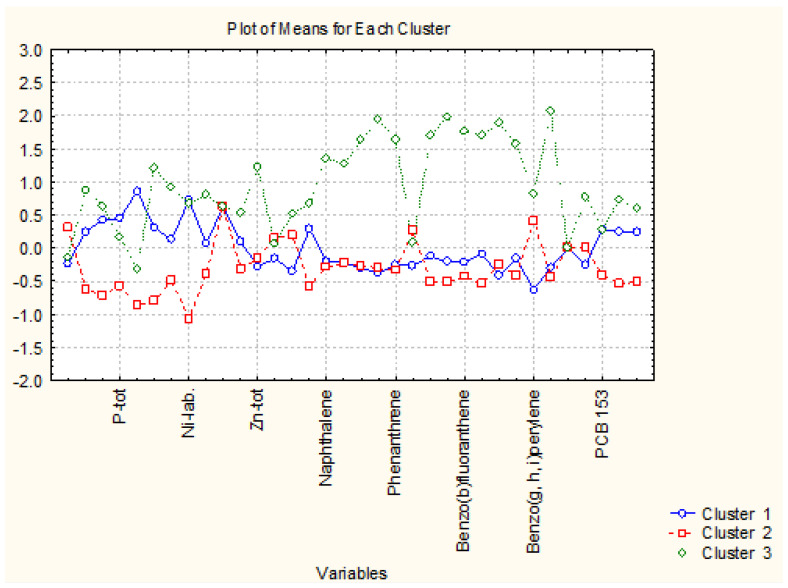
Plot of means for each variable for each identified cluster in zone C.

Figure 11 presents the average values for each variable for each identified 3 cluster. It is possible to find the specific parameters responsible for cluster formation.

Cluster 1 with 12 members out of a total of 20 is characterized by intermediate levels of almost all parameters. Cluster 2 (6 members out of a total of 20) is described by the lowest levels of all descriptors and it could be assumed that it is relatively free of pollution events. For comparison, the area of zone C populated by members of cluster 1 shows higher levels of metal pollution which is an indication of higher industrial pollution. The levels for PAHs and PCBs for clusters 1 and 2 are almost equal, which is proof of similar levels of impact from organic wastes. The two sites included in cluster 3 (rather outliers than typical sediment sites) are most seriously polluted by industrial and organic anthropogenic wastes as indicated by the average values for almost all parameters. Factor analysis data is shown in the Supplementary Materials—Table 4 for the obtained factor loadings for zone C. 

Four latent factors explain over 65% of the total variance of the system. The first latent factor, which explains over 24% of the total variance, includes high factor loadings for most of the PAH’s species and could be conditionally named “PAHs pollution factor”. The contrast between zones A and B is the presence of two PCB pollutants in factor 1, which suggests the presence of mixed sources of pollution determining the sediment quality in zone C.

Latent factor 2 structure confirms the assumption for the special processes in sediment formation in zone C since it represents another specific composition of variables with high factor loadings (representing all types of studied pollutants—N-tot as a nutrient, the mobile forms of Zn and nickel as metal pollutants, anthracene (PAH representative) and a sum of PCBs as representative of PCB pollutants). Further, loss of ignition belongs to factor 2. Thus, this factor could be conditionally named “complex pollution factor” (obviously typical for zone C only).

Latent factor 3 (nearly 14% explanation of the total variance) also shows complex and mixed character—most of the metal pollutants are mixed with the PAHs acenaphthene and fluorine—complex metal pollution factor”.

The last latent factor, 4, with the lowest percentage of variance explanation, also indicates metal pollution effect by the mobile form of Cr negatively correlated to the total forms of Cu and Ni. The P-tot is also included in this latent factor indicating the impact of the nutrient species. 

**Figure 12:**
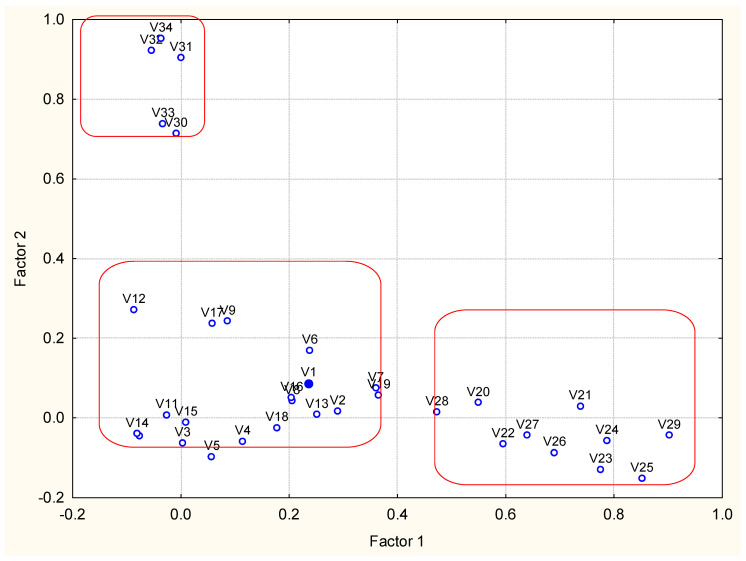
Biplot of factor loadings (Factor 1 vs. Factor 2 plane).

In general, the structure of the determined latent factors of zone C (Figure 12) illustrates that the dynamic conditions of polluting events in this zone are very different compared to zones A and B.

Partitioning of all sediment samples from zones A, B and C.

Set dimension [174 × 34]

Cluster analysis

**Figure 13:**
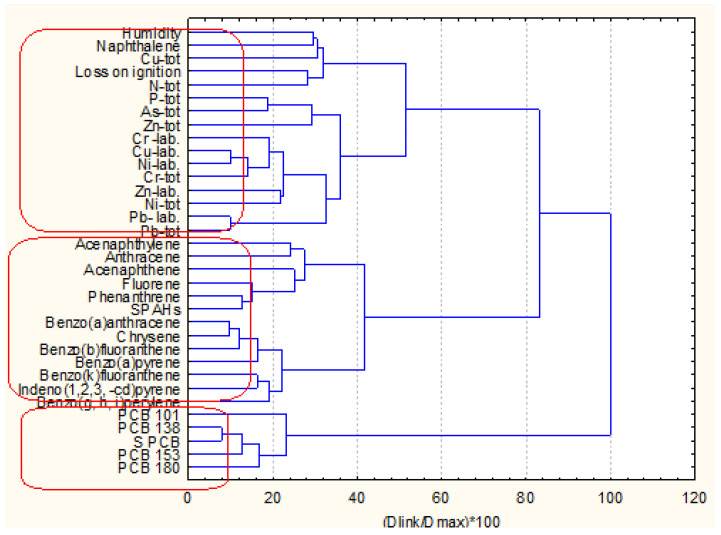
Hierarchical dendrogram for clustering of 34 variables, all zones.

**Figure 14:**
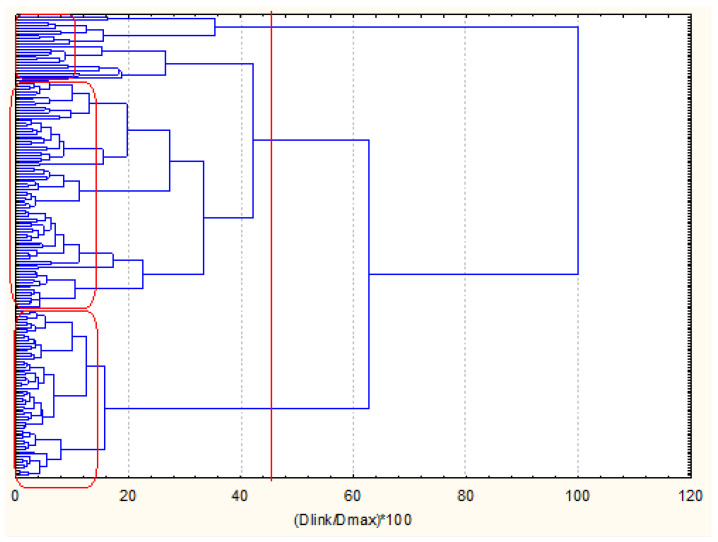
Hierarchical dendrogram for clustering of 174 objects from all three zones.

In Figure 13 and Figure 14, the hierarchical dendrograms for clustering, respectively, of variables and objects are shown. 

For the linkage of the variables, three major clusters are similar. They represent the close relationship between different groups of pollutants—cluster of PAHs, cluster of PCBs and cluster of metal pollutants. The metal pollutants cluster includes additionally the variables nutrients (N-tot and P-tot). 

The three clusters correspond to the three zone studies as discussed above. 

**Figure 15:**
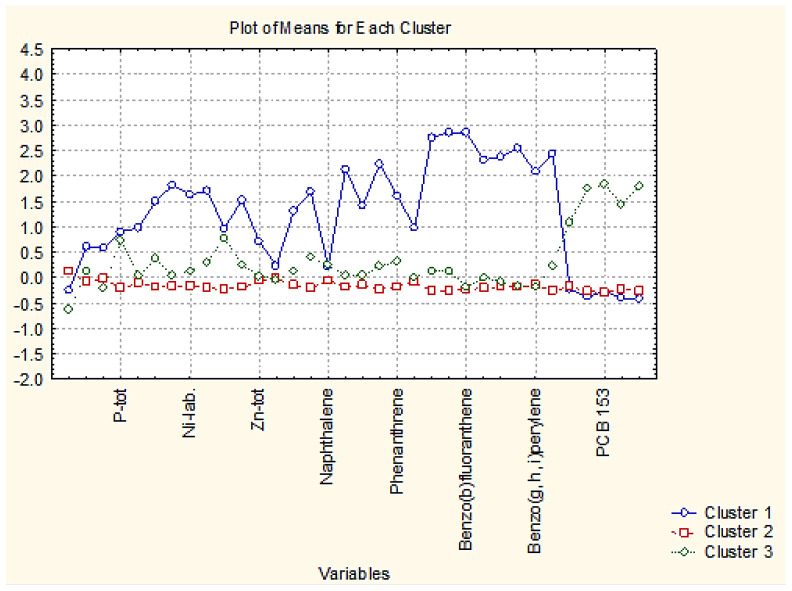
Plot of averages for each variable for each identified cluster (zone of sampling).

In Figure 15, the average values for each variable for each identified cluster are presented. Thus, it becomes possible to determine the specific variables specific for each of the clusters. A careful consideration of the members of each cluster shows that cluster 1 with 18 members includes dominantly sampling locations from zone C, cluster 2 with 115 members includes dominantly sediment samples from zone B and cluster 3 (41 members) corresponds to the sampling sites from zone A.

It is evident from the plot in Figure 15 that cluster 1 (zone C as a whole entity with anthropogenic and industrial pollution impact) is characterized by the highest levels of metal pollutants and PAHs but low levels of PCBs. This is indirect proof of the impact of specific industrial and anthropogenic wastes.

Cluster 2 (corresponding to recreation zone B) is obviously the cleanest area along the Varna gulf with the lowest levels of all variables included in the study. 

Cluster 3 (with dominant membership of sites from industrial zone A) shows an intermediate response to the pollution impact. Characteristic of this cluster is the highest levels of PCB pollutants and higher levels of pollutants as compared to the recreational zone.

It could be concluded that both approaches of intelligent data interpretation complete each other with comparable results both for the analysis of the single zone with quantitative determination of the pollution sources within each zone and for the overall analysis of all data available.

## 3. Materials and Methods

### 3.1. Sediment Sampling and Analysis

The sampling and sample analysis was performed by members of the Institute of Oceanology, Bulgarian Academy of Sciences, Varna, Bulgaria, in the summer of 2017. 

174 coastal sediment samples were collected during the drilling campaign in depth between 1 and 6 m for three conditional zones of the Varna gulf coastal line (see Figure 1): zone A (industrially impacted zone), zone B (recreational beach area), and zone C (impacted by anthropogenic and industrial wastes).

The samples were tested using:

- gas chromatography coupled to mass spectrometry for polyaromatic hydrocarbons (PAHs) and polychlorinated biphenyl congeners (PCBs).
- inductively coupled plasma—atomic absorption spectrometry (ICP-AAS) for metals as mobile and total forms (Cu, Zn, Ni, Cr), electrothermal AAS for Pb, and hybrid generation AAS for As.

The total amount of phosphorus was determined as orthophosphate after treatment of the sample with perchloric acid and the application of the ascorbic acid reduction method. Total nitrogen was determined by the Kjeldahl method. Humidity was measured by standard measurement of weight loss after drying and loss of ignition by weight loss after burning away the organic matter. 

All methods mentioned are well known and standardized for routine laboratory use [29].

### 3.2. Multivariate Statistical Methods

The methods of the intelligent analysis used in the present study are well known and thoroughly described [30,31]: hierarchical and non-hierarchical (K-means) clustering, principal components (factor) analysis, and Thurston–Spengler [32] apportionment procedure. The combined use of all these approaches is widely used for environmental studies for detecting patterns of similarity between variables and/or objects of the studied system, which is of help in better data interpretation and modeling.

Hierarchical clustering is an unsupervised chemometric method for revealing groups of closely linked objects (variables) within a large data set. Several simple steps are necessary to solve the task of clustering:

Standardization of the raw data (z-transform) to overcome problems with different dimensionality of the members of the set.

Calculation of the similarity between the objects of interest (squared Euclidean distance in the present study).

Choice of the mode of linkage between the objects (Ward’s method of linkage) and graphical presentation of the linkage results in a tree-like diagram called a dendrogram.

In the final step, the statistical significance of the clusters is determined using Sneath’s index (1/3 or 2/3 Dmax).

K-means non-hierarchical clustering is a supervised chemometric method where the number of clusters is a priori determined by a reasonable theoretical assumption (hypothesis) or expert opinion. 

As a chemometrics approach, principal components analysis (factor analysis) is a typical display method, which ensures the reduction in the size of the space of the variables. The substitution of a great number of correlated parameters by a small number of independent factors (principal components) is a useful tool for data interpretation and data structure explanation.

The apportionment modeling uses principal components analysis as a first step of the data analysis (calculation of the absolute principal components scores APCS) followed by multiple regression on the principal components obtained in the first step. Thus, an apportioning of the contribution of each possible source (natural or anthropogenic) on the mass content of each variable could be estimated without direct measurement of the real emissions. A full description of the apportionment regression modeling is presented in [31]. All statistical calculations are performed using the software package STATISTICA 7.0 (Palo Alto, CA, USA) [33].

## 4. Conclusions

The present study has shown that the sediment quality in a large section of the Gulf of Varna, Bulgaria depends mainly on three types of pollution sources—industrial and anthropogenic pollution impact related to metal pollutants, PAHs, and PCB in the environment under surveillance. Since the organization of the research included industrial (zone A), recreational (zone B), and mixed anthropogenic and industrial wastes (zone C), it was significant to identify the pollution sources in all affected zones and to determine the contribution of each identified source in the formation of the total concentrations of the pollutant species. The polluting sources for all three zones are generally similar, with slight deviations. The recreation zone has the lowest pollution levels, followed by the industrial zone and then the mixed anthropogenic and industrial wasted zone. 

In the text, some known pollution sources in the area are mentioned (soda plant, cement work, glassware plant, sewage system without waste water treatment plant, and highway traffic). However, this information is rather qualitative and cannot be used for quantitative validation of the regression models. There is a lack of quantitative assessment for the individual contribution of the real existing pollution source to the total pollution of the marine sediments and the present study is a first attempt to quantify to some extent the source contributions. 

By using the intelligent analysis of the data, we were able to define properly the discrepancy of the groups of similar objects of sediment sampling in each zone studied, and in summary, we were able to deduce the reasons for the external partitioning of the zones among themselves. The results obtained could be useful for solutions in valuing sediment quality and by means of the sediment analysis data as specific indicators for the overall ecological status of the area of interest.

## Figures and Tables

**Table 1:** Coastal sehdiment samples and analyzed species.

Area of Study	Input Data Set [Cases/ Variables]	Physicochem. Parameters	Mobile Form of Metals	Total Content of Metals and Nutrients	PAHs	PCBs
*Zone A* Anthropogen. impact	[49 × 34]	Humidity Loss of ignition	Cr-lab Zn-lab Cu-lab Ni-lab Pb-lab	N-tot P-tot As-tot Cr-tot Zn-tot Cu-tot Ni-tot Pb-tot	Naphtalene Acenaphtylene Acenaphtene Fluorene Phenanthrene Chrysene Anthracene Benzo(a)anthracene Benzo(b)fluoranthrene Benzo(k)fluoranthrene Benzo(a)pyrene Indeno(1,2,3-cd)pyrene Benzo(g,h,i)perylene ∑PAHs	PCB101 PCB138 PCB153 PCB180 ∑PCBs
*Zone B* recreational	[105 × 34]	Humidity Loss of ignition	Cr-lab Zn-lab Cu-lab Ni-lab Pb-lab	N-tot P-tot As-tot Cr-tot Zn-tot Cu-tot Ni-tot Pb-tot	Naphtalene Acenaphtylene Acenaphtene Fluorene Phenanthrene Chrysene Anthracene Benzo(a)anthracene Benzo(b)fluoranthrene Benzo(k)fluoranthrene Benzo(a)pyrene Indeno(1,2,3-cd)pyrene Benzo(g,h,i)perylene ∑PAHs	PCB101 PCB138 PCB153 PCB180 ∑PCBs
*Zone C* Industrial and domestic wastes impact	[20 × 34]	Humidity Loss of ignition	Cr-lab Zn-lab Cu-lab Ni-lab Pb-lab	N-tot P-tot As-tot Cr-tot Zn-tot Cu-tot Ni-tot Pb-tot	Naphtalene Acenaphtylene Acenaphtene Fluorene Phenanthrene Chrysene Anthracene Benzo(a)anthracene Benzo(b)fluoranthrene Benzo(k)fluoranthrene Benzo(a)pyrene Indeno(1,2,3-cd)pyrene Benzo(g,h,i)perylene ∑PAHs	PCB101 PCB138 PCB153 PCB180 ∑PCBs

**Table 2:** Source apportionment zone A.

Variables	Intercept Unidentified Sources %	F1% PAH Pollution Source	F2% Metal Pollution Source	F3% PCB Pollution Source	F4% Nutrient Pollution Source	R^2^ Model Fit Measure
N-tot						
	34.7	-	-	-	65.3	0.81
P-tot	29.0	12.2	44.6	-	14.2	0.87
Cr-lab.		14.3	35.7	13.2	-	0.79
	36.8	9.2	46.1	7.7	-	0.86
Zn-lab.	37.0	5.1	63.3	5.2	3.1	0.81
		2.8	49.5	3.4	-	0.77
Cu-lab.	23.3	7.9	51.1	6.2	-	0.81
Ni-lab.	44.3	9.2	64.1	4.7	-	0.79
Pb-lab.	34.8	-	74.1	2.2	-	0.76
As-tot	22.0	3.1	51.2	2.8	1.2	0.73
Cr-tot	23.7	2.5	49.7	2.1	-	0.75
Zn-tot	42.7	-	64.0	-	-	0.77
Cu-tot	45.7	1.3	72.1	-	-	0.83
Ni-tot	36.9	12.2	44.6	-	14.2	0.87
Pb-tot	26.6	14.3	35.7	13.2	-	0.79
Sum PAHs	17.6	82.4	-	-	-	0.84
Sum PCBs	22.7	-	-	77.3	-	0.87

Note: The performance of FA or PCA with determination of the factor loadings for each variable allows us to present conditional names of the factors in consideration. It is not an a priori act, it is a posteriori definition based on the factor loadings values.

**Table 3:** Source apportionment zone B.

Variables	Intercept Unidentified Sources %	F1% PAH Pollution Source	F2% Metal Pollution Source	F3% PCB Pollution Source	F4% Nutrient Pollution Source	R^2^ Model Fit Measure
N-tot						
			unsatisfactory	model		
P-tot			unsatisfactory	model		
Cr-lab.			unsatisfactory	model		
Zn-lab.	23.5	8.3		68.2		0.77
	11.8	12.1		70.1		0.81
Cu-lab.	24.4	10.7		64.9		0.84
Ni-lab.	19.1	14.2		66.7		0.78
Pb-lab.	23.5	8.3		68.2		0.77
As-tot		unsatisfactory	model			unsatisfactory
Cr-tot	18.5		72.4	9.1	0.79	
Zn-tot		unsatisfactory	model			unsatisfactory
Cu-tot		unsatisfactory	model			unsatisfactory
Ni-tot		unsatisfactory	model			unsatisfactory
Pb-tot	18.2	5.6	62.3	7.1	0.84	5.6
Sum PAHs	17.8		10.1	28.9	0.81	
Sum PCBs	20.1	79.9			0.79	79.9

**Table 4:** Source apportionment models for zone C.

Variables	Intercept Unidentified Sources %	F1% PAH Pollution Source	F2% Metal Pollution Source	F3% PCB Pollution Source	F4% Nutrient Pollution Source	R^2^ Model Fit Measure
N-tot		8.4	47.2	3.2	4.4	0.84
	36.8	-	-	11.4	54.2	0.81
P-tot	34.4	-	9.9	-	55.7	0.79
Cr-lab.	34.4	-	61.1	10.4	9.2	0.79
	20.3	7.2	12.3	58.9	-	0.81
Zn-lab.	22.6	3.6	63.2	10.1	6.8	0.84
	16.3	3.9	3.7	55.4	4.1	0.75
Cu-lab.	32.9	-	10.1	46.2	-	0.73
Ni-lab.	43.7	-	11.1	54.2	-	0.75
Pb-lab.	34.7	-	-	65.1	9.1	0.79
As-tot	25.8	-	20.2	-	71.3	0.84
Cr-tot	8.5	-	-	11.3	59.9	0.81
Zn-tot	28.8	-	6.2	67.7	7.1	0.83
Cu-tot	28.0	54.8	-	30.6	-	0.81
Ni-tot	14.6	25.7	60.3	-	-	0.79
Pb-tot	14.0	8.4	47.2	3.2	4.4	0.84
Sum PAHs	36.8	-	-	11.4	54.2	0.81
Sum PCBs	34.4	-	9.9	-	55.7	0.79

## Data Availability

Data available in the Supplementary Materials.

## Supplementary Materials

The following supporting information can be downloaded at: https://www.mdpi.com/article/10.3390/molecules27196539/s1, Tables S1, S2 and S4: Factor loadings; Table S3: Decoded variables tables.

## Sample Availability

Samples of the compounds are not available from the authors.
