# Supplementary material for: Developing an Intelligent Data Analysis Approach for Marine Sediments
Source: Molecules. 2022 Oct 3;27(19):6539. doi: 10.3390/molecules27196539 (PMC9573499; doi:10.3390/molecules27196539)
Supplement: Supplementary file 1 [file molecules-27-06539-s001.zip › molecules-1890023-supplementary.pdf]

Supplementary Materials:

Developing an intelligent data analysis approach for marine sediments

Miroslava Nedyalkova [1] and Vasil Simeonov [2, *]

[1] Department of Inorganic Chemistry, Faculty of Chemistry and Pharmacy, University of Sofia "St. Kl.

Ohridski", 1164 Sofia, Bulgaria

[2] Department of Analytical Chemistry, Faculty of Chemistry and Pharmacy, University of Sofia "St. Kl.

Ohridski", 1164 Sofia, Bulgaria

* Correspondence vsimeonov@chem.uni-sofia.bg.

Table S1. Factor loadings

Factor Loadings (Varimax normalized) Extraction: Principal components (Marked loadings are significant)				
**Variables**	**Factor 1**	**Factor 2**	**Factor 3**	**Factor 4**
**Humidity**	0.025	**0.483**	-0.234	**0.453**
**Loss on ignition**	0.322	**0.635**	0.091	-0.425
**N-tot**	0.072	0.160	-0.130	**0.561**
**P-tot**	0.118	**0.634**	0.143	0.029
**Cr -lab.**	0.095	**0.916**	-0.020	-0.155
**Zn-lab.**	0.340	**0.828**	0.053	-0.104
**Cu-lab.**	0.163	**0.883**	-0.098	-0.162
**Ni-lab.**	0.194	**0.920**	-0.064	-0.174
**Pb- lab.**	0.491	**0.787**	-0.031	-0.024
**As-tot**	0.026	**0.709**	0.002	0.206
**Cr-tot**	0.234	**0.933**	-0.110	-0.048
**Zn-tot**	0.203	**0.805**	-0.066	-0.048
**Cu-tot**	0.056	**0.892**	-0.042	0.024

**Ni-tot**	0.093	**0.810**	-0.103	0.035
**Pb-tot**	0.383	**0.832**	-0.062	0.091
**Naphthalene**	0.074	0.066	0.382	0.598
**Acenaphthylene**	**0.690**	0.030	0.010	0.332
**Acenaphthene**	**0.646**	0.385	0.366	0.231
**Fluorene**	**0.881**	0.179	0.031	0.158
**Phenanthrene**	**0.728**	0.306	0.280	0.090
**Anthracene**	**0.773**	-0.083	0.022	0.243
**Benzo(a)anthracene**	**0.937**	0.211	0.026	-0.015
**Chrysene**	**0.872**	0.381	0.068	-0.140
**Benzo(b)fluoranthene**	**0.906**	0.268	-0.081	-0.220
**Benzo(k)fluoranthene**	**0.809**	0.142	-0.013	-0.176
**Benzo(a)pyrene**	**0.840**	0.105	-0.005	-0.292
**Indeno(1,2,3, -cd)pyrene**	**0.795**	0.236	-0.037	-0.319
**Benzo(g, h, i)perylene**	**0.869**	0.258	-0.068	-0.174
**SPAHs**	**0.859**	0.265	0.253	0.201
**PCB 101**	0.139	0.021	**0.790**	0.109
**PCB 138**	0.014	-0.056	**0.961**	0.140
**PCB 153**	0.099	-0.067	**0.951**	0.018
**PCB 180**	-0.042	-0.070	**0.900**	0.024
**S PCB**	0.027	-0.070	**0.982**	0.053
**Expl.Var %**	28.2	28.1	14.2	8.1

Table S2. Factor loadings

Factor Loadings (Varimax normalized):Principal components (Marked loadings are significant)				
**Variable**	**Factor1**	**Factor2**	**Factor3**	**Factor4**
**Humidity**	0.056	-0.013	0.294	0.198
**Loss on ignition**	0.074	0.037	0.322	0.205
**N-tot**	0.085	0.035	0.088	-0.294
**P-tot**	0.168	0.035	0.009	-0.077
**Cr -lab.**	0.133	0.047	0.235	-0.405
**Zn-lab.**	-0.027	-0.100	**0.691**	0.090
**Cu-lab.**	0.119	-0.027	**0.712**	-0.024
**Ni-lab.**	0.054	0.022	**0.709**	-0.383
**Pb- lab.**	-0.169	-0.107	**0.677**	-0.040

**As-tot**	-0.028	-0.007	0.191	-0.229
**Cr-tot**	0.058	0.054	**0.711**	-0.166
**Zn-tot**	-0.125	0.070	0.077	-0.021
**Cu-tot**	-0.019	0.015	-0.002	0.058
**Ni-tot**	0.001	0.032	0.484	0.011
**Pb-tot**	-0.167	-0.109	**0.639**	-0.177
**Naphthalene**	0.013	0.067	0.207	0.191
**Acenaphthylene**	**0.655**	0.037	-0.128	0.057
**Acenaphthene**	0.015	-0.011	0.048	**0.826**
**Fluorene**	0.151	0.015	0.146	**0.899**
**Phenanthrene**	0.433	-0.013	0.184	**0.747**
**Anthracene**	0.379	0.070	-0.031	**0.772**
**Benzo(a)anthracene**	**0.895**	0.049	-0.033	0.213
**Chrysene**	**0.920**	-0.028	0.018	0.134
**Benzo(b)fluoranthene**	**0.910**	0.094	0.217	0.061
**Benzo(k)fluoranthene**	**0.678**	0.041	0.245	0.031
**Benzo(a)pyrene**	**0.768**	0.027	-0.194	-0.067
**Indeno(1,2,3, -cd)pyrene**	**0.720**	-0.007	0.302	-0.182
**Benzo(g, h, i)perylene**	0.276	0.039	**0.610**	0.028
**SPAHs**	**0.786**	0.041	0.253	0.594
**PCB 101**	-0.028	**-0.713**	0.059	-0.028
**PCB 138**	-0.063	**-0.905**	0.037	0.090
**PCB 153**	-0.062	**-0.938**	0.047	-0.065
**PCB 180**	0.008	**-0.774**	-0.025	-0.091
**S PCB**	-0.048	**-0.973**	0.012	-0.014
**Expl.Var %**	25.8	18.3	11.1	10.1

Table S3. Decoded variables tables

**Variables**	**Code**
**Humidity**	**V1**
**Loss on ignition**	**V2**
**N-tot**	**V3**

**P-tot**	**V4**
**Cr -lab.**	**V5**
**Zn-lab.**	**V6**
**Cu-lab.**	**V7**
**Ni-lab.**	**V8**
**Pb- lab.**	**V9**
**As-tot**	**V10**
**Cr-tot**	**V11**
**Zn-tot**	**V12**
**Cu-tot**	**V13**
**Ni-tot**	**V14**
**Pb-tot**	**V15**
**Naphthalene**	**V16**
**Acenaphthylene**	**V17**
**Acenaphthene**	**V18**
**Fluorene**	**V19**
**Phenanthrene**	**V20**
**Anthracene**	**V21**
**Benzo(a)anthracene**	**V22**
**Chrysene**	**V23**
**Benzo(b)fluoranthene**	**V24**
**Benzo(k)fluoranthene**	**V25**
**Benzo(a)pyrene**	**V26**
**Indeno(1,2,3, -cd)pyrene**	**V27**
**Benzo(g, h, i)perylene**	**V28**
**SPAHs**	**V29**
**PCB 101**	**V30**
**PCB 138**	**V31**
**PCB 153**	**V32**
**PCB 180**	**V33**
**S PCB**	**V34**

Descriptive Statistics (Spreadsheet1)					
	**Valid N**	**Mean**	**Minimum**	**Maximum**	**Std.Dev.**
**Humidity**	174	17.7037	4.3800	27.000	3.7995
**Loss on ignition**	174	0.3138	0.0400	1.770	0.1868
**N-tot**	174	791.9195	103.0000	2926.000	453.3002
**P-tot**	174	267.4551	144.9700	984.340	109.3148
**Cr -lab.**	174	0.7511	0.1250	4.383	0.4805
**Zn-lab.**	174	2.6744	0.7631	12.085	1.2883
**Cu-lab.**	174	0.4495	0.1250	5.596	0.5566
**Ni-lab.**	174	0.4336	0.1250	5.383	0.4745
**Pb- lab.**	174	1.8978	0.6517	5.503	0.6969
**As-tot**	174	0.9731	0.6250	5.050	0.6861
**Cr-tot**	174	2.6900	1.2400	19.540	1.7015
**Zn-tot**	174	8.7082	2.3710	59.330	6.4428
**Cu-tot**	174	0.9081	0.1250	45.000	3.4560
**Ni-tot**	174	1.4799	0.3808	11.820	1.2644
**Pb-tot**	174	2.5138	0.9624	7.404	0.8271
**Naphthalene**	174	0.0017	0.0005	0.053	0.0052
**Acenaphthylene**	174	0.0006	0.0005	0.002	0.0002
**Acenaphthene**	174	0.0009	0.0005	0.012	0.0010
**Fluorene**	174	0.0014	0.0005	0.007	0.0012
**Phenanthrene**	174	0.0028	0.0005	0.010	0.0019
**Anthracene**	174	0.0007	0.0005	0.006	0.0005
**Benzo(a)anthracene**	174	0.0010	0.0005	0.006	0.0010
**Chrysene**	174	0.0011	0.0005	0.005	0.0009
**Benzo(b)fluoranthene**	174	0.0020	0.0005	0.013	0.0020
**Benzo(k)fluoranthene**	174	0.0007	0.0005	0.003	0.0004
**Benzo(a)pyrene**	174	0.0006	0.0005	0.003	0.0003
**Indeno(1,2,3, -cd)pyrene**	174	0.0012	0.0005	0.009	0.0013
**PCB 28**	174	0.0009	0.0005	0.005	0.0008
**PCB 52**	174	0.0179	0.0005	0.086	0.0163
**PCB 101**	174	0.0001	0.0001	0.000	0.0000

**PCB 138**	174	0.0002	0.0001	0.001	0.0002
**PCB 153**	174	0.0001	0.0001	0.001	0.0001
**PCB 180**	174	0.0002	0.0001	0.001	0.0002
**S PCB**	174	0.0006	0.0001	0.004	0.0006

Table S4. Factor loadings for zone C

Factor Loadings (Varimax normalized)) Extraction: Principal components (Marked loadings are significant				
**Variables**	**Factor1**	**Factor2**	**Factor3**	**Factor4**
**Humidity**	-0.228	-0.185	-0.078	-0.019
**Loss on ignition**	0.102	**0.720**	0.113	0.210
**N-tot**	0.177	**0.797**	0.131	-0.088
**P-tot**	-0.026	0.047	0.423	**-0.729**
**Cr -lab.**	0.014	0.473	0.020	**-0.795**
**Zn-lab.**	0.186	**0.745**	0.589	0.194
**Cu-lab.**	0.188	0.354	**0.742**	-0.009
**Ni-lab.**	0.168	**0.724**	0.464	-0.428
**As-tot**	0.123	0.231	**0.698**	0.021
**Pb- lab.**	0.148	0.122	**0.766**	-0.127
**Cr-tot**	0.081	0.109	**0.698**	-0.070
**Zn-tot**	0.100	-0.044	**0.683**	0.450
**Cu-tot**	-0.062	0.302	0.188	**0.684**
**Ni-tot**	-0.095	0.066	0.374	**0.750**
**Pb-tot**	0.058	0.286	**0.770**	-0.221
**Naphthalene**	0.046	-0.153	**0.701**	0.250
**Acenaphthylene**	**0.777**	0.060	-0.139	-0.297
**Acenaphthene**	0.241	-0.180	**0.701**	0.253
**Fluorene**	0.628	-0.191	**0.700**	0.137
**Phenanthrene**	**0.715**	-0.110	0.507	0.010
**Anthracene**	0.242	**0.683**	0.178	-0.033
**Benzo(a)anthracene**	**0.847**	0.092	0.335	-0.234

**Chrysene**	**0.800**	0.159	0.471	0.012
**Benzo(b)fluoranthene**	**0.736**	0.206	0.511	0.165
**Benzo(k)fluoranthene**	**0.846**	0.397	0.143	-0.138
**Benzo(a)pyrene**	**0.781**	0.179	0.351	0.309
**Indeno(1,2,3, -cd)pyrene**	**0.686**	0.399	0.396	0.175
**Dibenzo(a, h)anthracene**	**0.777**	0.060	-0.139	-0.297
**Benzo(g, h, i)perylene**	**0.625**	-0.302	0.033	0.320
**SPAHs**	**0.725**	0.034	0.629	0.169
**PCB 138**	**0.627**	0.373	-0.344	0.368
**PCB 153**	0.102	0.419	0.195	-0.139
**PCB 180**	**0.687**	0.555	-0.307	-0.213
**S PCB**	0.466	**0.742**	-0.226	-0.028
**Expl.Var %**	24.1	19.7	13.8	10.6
